# Targeted Alteration of Antibody-Based Immunodominance Enhances the Heterosubtypic Immunity of an Experimental PCV2 Vaccine

**DOI:** 10.3390/vaccines8030506

**Published:** 2020-09-04

**Authors:** AGM Rakibuzzaman, Oleksandr Kolyvushko, Gagandeep Singh, Peter Nara, Pablo Piñeyro, Estelle Leclerc, Angela Pillatzki, Sheela Ramamoorthy

**Affiliations:** 1 Department of Microbiological Sciences, North Dakota State University, Fargo, ND 58102, USA; agm.rakibuzzaman@ndsu.edu (A.R.); kolyvushko.oleksandr@gmail.com (O.K.); gagandeep.singh@ndsu.edu (G.S.); 2 Keystone Bio Inc., St. Louis, MO 63110, USA; nara@keystonebio.com; 3 College of Veterinary Medicine, Iowa State University, Ames, IA 50011, USA; pablop@iastate.edu; 4 Department of Pharmaceutical Sciences, College of Health Professions, North Dakota State University, Fargo, ND 58102, USA; estelle.leclerc@ndsu.edu; 5 Animal Disease Research and Diagnostic Laboratory, South Dakota State University, Brookings, SD 57007, USA; angela.pillatzki@sdstate.edu

**Keywords:** vaccine, porcine circovirus, PCV2, decoy epitope, antibody, virus neutralization, mutation

## Abstract

Despite the availability of commercial vaccines which can effectively prevent clinical signs, porcine circovirus type 2 (PCV2) continues to remain an economically important swine virus, as strain drift, followed by displacement of new subtypes, occurs periodically. We had previously determined that the early antibody responses to the PCV2 capsid protein in infected pigs map to immunodominant but non-protective, linear B cell epitopes. In this study, two of the previously identified immunodominant epitopes were mutated in the backbone of a PCV2b infectious clone, to rationally restructure the immunogenic capsid protein. The rescued virus was used to immunize 3-week-old weanling piglets, followed by challenge with a virulent heterologous PCV2d strain. As expected, immunodominant antibody responses to the targeted epitopes were abrogated in vaccinated pigs, while a broadening of the virus neutralization responses was detected. Vaccinated pigs were completely protected against challenge viral replication, had reduced microscopic lesions in lymphoid organs and gained significantly more body weight when compared to unvaccinated pigs. Thus, the experimental PCV2 vaccine developed was highly effective against challenge, and, if adopted commercially, can potentially slow down or eliminate new strain creation.

## 1. Introduction

Porcine circovirus type 2 (PCV2) is a small, single-stranded DNA virus which belongs to the *circoviridae* family. It is an economically important swine virus which causes post-weaning multi-systemic wasting syndrome (PMWS) and lymphadenopathy in weanling piglets, along with a range of clinical signs including jaundice, nephropathy, reproductive and respiratory disorders, collectively known as porcine circovirus associated diseases or PCVAD [1]. Several commercial vaccines against PCV2 are available and commonly deployed in the field. They are very effective in preventing clinical signs of PCV2 and in reducing economic losses associated with PCV2 infection. However, they do not prevent transmission or shedding of PCV2. Most of the commercial vaccines continue to target the first discovered PCV2 subtype: PCV2a. Commercial vaccines contain either the whole inactivated virus, inactivated chimeric PCV1-2a virus preparations or subunits of the PCV2a capsid protein. After the introduction of commercial vaccines, the initially predominating field subtype, designated PCV2a, was replaced by PCV2b and, more recently, by PCV2d. While serological cross-reactivity between the subtypes and cross-protection due to vaccination is observed, it is possible that selection pressure induced by immunity developed against commercial vaccines could be driving viral evolution in the field [1,2,3].

The approximately 1700bp PCV2 genome encodes just two major proteins: the replicase and capsid proteins. The capsid protein is considered to be both necessary and sufficient for the prevention of PCV2, as the subunit vaccination with the capsid protein alone is effective at preventing the clinical signs of PCVAD. While the cell mediated immune response to PCV2 is not well studied, neutralizing antibody responses targeted to the capsid protein are considered to be critical for protection against PCV2 [1,2]. Strong binding Ab responses to PCV2 can be detected as early as 7 days post-infection in naturally or experimentally infected pigs. However, neutralizing Ab responses, which correlate with a reduction in viremia, are not detected until later in the course of infection [4]. Immuno-dominance, the phenomenon by which the immune system preferentially mounts responses to selected antigens, or epitopes within antigens, is an effective immuno-subversion mechanism for pathogens, and a well-established confounding factor in the development of effective vaccines [5]. The delayed production of neutralizing Ab responses, coupled with the periodical emergence of new PCV2 subtypes following vaccination suggests that antibody based immunodominance plays an important role in PCV2 pathogenesis and vaccine mediated protection. Currently, PCV2 vaccines are extensively deployed in pork production units. Based on diagnostic case submissions, it is estimated that a large percentage of production pigs also harbor the virus [1]. It has been suggested that lack of vaccine compliance due to improper storage or administration, and not a lack of cross-protection, is responsible for the shift in genotypes [6]. Therefore, the availability of tools to monitor vaccine compliance in the field can advance the control of PCV2 significantly.

In a previous study, we had explored the hypothesis that the early Ab responses in PCV2 infected pigs would be directed towards non-protective epitopes in the PCV2 capsid protein. Using sequential anti-sera collected from infected pigs, and a panel of overlapping peptides spanning the PCV2 capsid protein, we identified three new linear immunodominant—but non-protective—regions of the PCV2 capsid protein [7]. We also confirmed the presence of a previously identified immuno-dominant decoy epitope [7,8,9]. In addition, we found that a majority of the Abs produced by vaccination mapped to the non-protective immunodominant epitopes identified in the study. Hence, the primary objective of this study was to introduce mutations in two of the previously identified non-protective epitopes to alter immunodominance patterns and evaluate the performance of the modified recombinant virus as a vaccine. The secondary objective of this study was to develop a marker vaccine against PCV2 by introducing an immunogenic foreign peptide in the vaccine construct, to enable monitoring of vaccine compliance.

Vaccination of pigs with the restructured PCV2b vaccine (rPCV2-Vac) encoding a marker, and challenge with the currently predominating heterologous PCV2d strain resulted in improved heterosubtypic virus neutralization responses, protection against tissue pathology, lack of viremia due to the challenge virus, improved weight gain and Ab responses specific to the marker. The strategy described in this manuscript provides insights into the mechanisms of vaccine-mediated protection against PCV2 with long-term implications for improving the control and prevention of PCV2.

## 2. Materials and Methods

### 2.1. Cells and Viruses

The PCV1 free porcine kidney cell line, PK-15N (005-TDV, National Veterinary Services Laboratory, Ames, IA, USA), was used to culture all PCV2 strains. An infectious clone of PCV2b strain 41,513 (GenBank accession number KR816332) was used as the backbone to develop the restructured PCV2 vaccine. An infectious clone of a heterologous PCV2d strain (GenBank accession number JX535296.1) was used to prepare the challenge virus [10]. For virus neutralization assays, PCV2a (AF264042.1), PCV2b (EU340258.1) and PCV2d (JX535296.1) infectious clones were used to generate virus stocks by transfection as described below.

### 2.2. Cloning of the Vaccine Construct

Using the infectious clone of PCV2b 41,513 as the backbone, two previously identified linear immuno-dominant, but non-protective epitopes in the immunogenic PCV2 capsid protein [7,8] were mutated. The capsid gene segment encoding the desired mutations was commercially synthesized and cloned into the backbone of PCV2b 41,513 by restriction digestion. To minimize the risk of producing a lethal mutation, selected amino acids in the linear decoy epitopes were replaced with other amino acids with a low penalty score on a point accepted mutation (PAM) matrix [11]; Epitope A124 ILDDNFVTKATALTYDPY 141 [7] was modified to 124 ILDDNFVNKSTALTYDPY 141 and epitope B166 VLDSTIDYFQPNNKR 180 [8] was modified to 166 VLDSTIDYFNPNNSR 180 (Table S1, Figures S1 and S2). The replacement of a threonine (T) with an asparagine (N) residue in epitope A resulted in the introduction of a putative N-linked glycosylation sequon (NxS) (Table S1). Epitope B naturally contained a predicted N-linked glycosylation site (Table S1) and was not altered for glycosylation properties. All mutations were validated by sequencing (Eurofin Genomic, Louisville, KY, USA). The vaccine construct is henceforth referred to as the re-structured PCV2 vaccine (rPCV2-Vac) throughout the manuscript.

### 2.3. Insertion of a Marker to Enable the Monitoring of Vaccine Compliance

**Figure 1:**
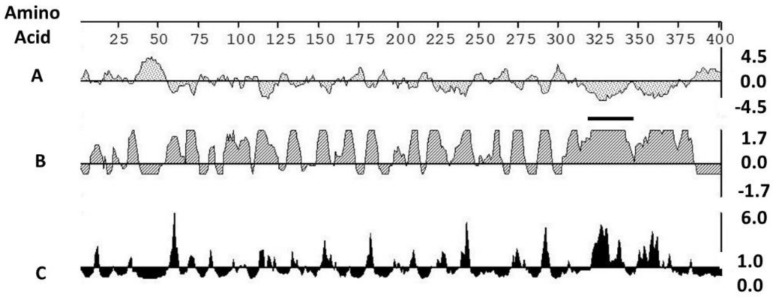
Selection of an immunogenic marker: Evaluation of the antigenicity of the *Neospora caninum* SRS2 protein by: **A**. Kyle Doolittle hydropathy plot—a negative value indicates hydrophilic residues; **B**. Jameson Wolfe antigenicity index; **C**. Emini surface probability plot. **B** and **C**: the height of the vertical bar is proportional to the predicted antigenicity of the sequence. **A** positive value indicates higher immunogenicity. Horizontal bar: amino acid positions in the sequence selected for analysis. Solid dark line: peptide sequence 324 QSSEKRDGEQVNKGKPP 348 selected as the marker in the vaccine construct.

To enable the monitoring of vaccine compliance using a serological assay, the vaccine construct was tagged with an immunogenic marker. *Neospora caninum* is an apicomplexan parasite which has not been detected in pigs [12]. A highly immunogenic segment of 18 amino acid length selected from the surface antigen-1 related sequence 2 (SRS2) protein (AAD04844.1) of *N. caninum* was selected following the in-silico prediction of antigenicity (Lasergene 11, Protean 13, DNASTAR, Madison, WI, USA) (Figure 1). The selected sequence was subjected to a protein blast to rule out possible serological cross reactivity with other swine related proteins. Amino acids 324 QSSEKRDGEQVNKGKPP 348 of the SRS2 protein, with an antigenicity index score of 1.7 (Figure 1), was inserted into 5′ end of the capsid gene of the rPCV2-Vac construct described above, as a separate transcriptional unit (Figure S2), using the Q5 mutagenesis kit (New England Biologicals, Ipswich, MA, USA), according to the manufacturer’s instructions.

### 2.4. Preparation of PCV2 Virus Cultures

The vaccine and challenge virus cultures, as well as the virus cultures required for the virus neutralization assay, were prepared by transfection of PK-15 cells [13], with some modifications. Briefly, the PCV2 genome was excised from the shuttle plasmid by restriction digestion and re-circularized with DNA ligase, unless dimerized infectious clones were available. For transfection, 12 µg of viral genomic DNA or plasmids containing the dimerized infectious clones [10,14] were diluted in Opti-MEM, mixed with 36 µL of TransIT-2020 (Mirus Bio, Madison, WI, USA), and incubated at room temperature for 30 min. After the incubation period, the mixture was overlaid on cell culture flasks (25 cm^2^, Corning, Tewksbury, MA, USA) containing 50% confluent monolayers of PK-15 cells and incubated at 37 °C in a CO_2_ incubator for 3h, followed by addition of Dulbecco’s Modified Eagle’s Medium (DMEM) with 2% fetal bovine serum and 1X penicillin-streptomycin. The flasks were frozen and thawed three times after 72 h of incubation. The rescued viruses were titrated by the TCID_50_ method. The stock cultures were stored at −80 °C until used.

### 2.5. Immunofluorescence Assay

As PCV2 does not produce cytopathic effects, replication of the PCV2 strains was visualized by an IFA as previously described [13]. Briefly, 50% confluent PK-15 monolayers grown in eight well chamber slides were either transfected as described above or infected with the virus cultures [15]. After 72 h of incubation in a CO_2_ incubator, the cells were fixed with a 1:1 mixture of methanol and acetone. The fixed cell sheets were stained with a PCV2 specific monoclonal antibody (Rural Technologies, Brookings, SD, USA) or *Neospora caninum* specific polyclonal antibody, followed by detection with a FITC-conjugated secondary antibody (KPL, SeraCare, Milford, MA, USA), and counter-staining with DAPI (Life Technologies, Carlsbad, CA, USA). The stained cells were evaluated for apple green nuclear fluorescence indicative of PCV2 replication or expression of the SRS2 marker tag.

### 2.6. In-Vitro Vaccine Stability

The rPCV2-Vac cultures rescued by transfection of PK-15 cells were serially passaged three times in PK-15 cells. Virus titers were compared against the wildtype virus. The construct was sequenced to verify the stability of the mutations.

### 2.7. Vaccination and Challenge of Piglets

All procedures pertaining to animal experimentation were carried out with the approval and oversight of the Institutional Animal Care and Use Committee (IACUC) and Institutional Biosafety Committee (IBC) regulations of N. Dakota (NDSU) and S. Dakota State Universities (SDSU). Twenty-seven 3–4-week-old piglets, which were serologically and PCR negative for PCV2 and other major swine pathogens, such as PRRSV, SIV and *Mycoplasma* sp., were divided into three groups of 9 pigs each. Group I was administered PBS, group II were administered a commercial, inactivated PCV2 vaccine as per label instructions (2 mL, intramuscular), and group III were inoculated with the rPCV2-Vac at 10^4^TCID_50_/mL, 2 mL intramuscular and 2 mL intranasally. Although the exact details regarding the antigen dose and formulation of the commercial vaccine are not publicly available, a commercial vaccine was selected as a control to represent current industry standards. The vaccine used consisted of a PCV1-2a chimeric virus, wherein the PCV2a capsid gene was cloned in the backbone of the non-pathogenic PCV1 [16], followed by inactivation and formulated with squalene as an adjuvant (Fostera^®^ PCV MetaStim^®^, Zoetis, Inc., Parsippany, NJ 07054, USA). Vaccinated pigs were boosted with the same dose and route on day 14 post-vaccination (DPV). On DPV 28 post or day 0 post-challenge (DPC), all study animals were challenged with a heterologous PCV2d strain at 10^4^TCID_50_, 2 mL intramuscular and 2 mL intranasally. Two pigs per group were sacrificed prior to challenge to assess vaccine safety. Pigs were monitored daily for signs of porcine circovirus associated diseases (PCVAD), such as wasting, respiratory distress, jaundice, inappetence or diarrhea. Body weights were assessed on DPC 0, 9 and 21 (Figure S3). Serum samples were collected on day 0, and every 2 weeks thereafter to assess Ab responses. All animals were humanely euthanized on DPC 21 for evaluation of pathological lesions as described below.

### 2.8. Anti-PCV2 IgG Responses

The measurement of binding IgG responses to PCV2 in vaccinated pigs was achieved with a commercial PCV2 Enzyme linked immunosorbent assay ELISA kit (Ingezim Circovirus IgG kit, Ingenasa, Madrid, Spain), at the Iowa State University Veterinary Diagnostic Laboratory, following their standard operating procedures and the manufacturer’s instructions. Sample to positive control (S/P) ratios produced as the assay output were used for further analysis of the data.

### 2.9. Virus Neutralizing Antibody Responses

Functional antibody responses against the homologous PCV2b subtype and heterologous PCV2a and PCV2d subtypes were measured by a rapid fluorescence focus neutralization (FFN) assay, essentially as described before [7], except that the virus cultures were adjusted to 30–40 fluorescent focus units (FFU)/100 µL for consistent enumeration. Virus replication was assessed by an IFA, as described above. Four replicate values of the DPV 28 sera were obtained and used for analysis. The titers were expressed as the percentage reduction in viral replication compared to the virus only control, which was not treated with serum.

### 2.10. Antibody Responses to the Mutated Epitopes

The abrogation of the immunodominant Ab response to the selected epitopes in vaccinated pigs was assessed by surface plasmon resonance on a Reichert SR7500DC instrument (Reichert Technologies, Buffalo, NY, USA). Biotinylated peptides encoding the wildtype peptide sequences of epitopes A and B described above were commercially synthesized (Biomatik, Wilmington, DE, USA). Pooled sera collected at DPV 28 from the three treatment groups and from archived sera collected from PCV2b infected pigs [17] (provided by X. J. Meng, Virginia Tech, Blacksburg, VA, USA) were used to purify IgG using a commercial kit (Melon gel IgG purification kit, Thermo Fisher, Waltham, MA, USA). The biotinylated peptides were immobilized on streptavidin coated carboxymethyl dextran sensor chips (Reichert Technologies, USA) by injecting 0.16 μg/μL peptide solution over the sensor chip at a flow rate of 25 μL/min. After an increase of about 300 µRU was observed, indicating immobilization of each peptide had occurred, the purified IgGs for the experimental groups were injected over the flow cells at a concentration of 20 µM in phosphate buffered saline with 0.005% Tween 20 (PBST), at a flow rate of 25 μL/min for 240 s. Binding of the IgGs to the peptides was assessed by the response in µ response units (µRU).

### 2.11. Antibody Responses to the Marker

The selected peptide from the *N. caninum* SRS2 protein was cloned into a bacterial expression vector (pETSumo Thermo Fisher Scientific, USA) using the Q5 site directed mutagenesis kit (New England Biologicals, Ipswich, MA, USA). The protein was expressed with a HIS tag and purified with by nickel affinity chromatography (His-spin protein miniprep, Zymo research, Irvine, CA, USA), following the manufacturer’s instructions. The identity of the purified protein was verified by Western blotting with an anti-HIS tag specific monoclonal Ab (Figure S3). The purified protein was used to coat ELISA plates, followed by washing with PBST and blocking (General block with 2% BSA, Immuno Chemistry Technologies, Bloomington, MN, USA) for 2 h at 37 °C. The blocked plates were washed with PBST. A 1:50 dilution of the test anti-sera was diluted in PBS with 2% BSA, added to the wells, and incubated for 2 h. The plates were then reacted with a 1:5000 dilution of anti-swine IgG conjugated to HPO (KPL, SeraCare, Milford, MA, USA), followed by addition of TMB substrate. The reaction was stopped with 1M HCl and plate was read at 450 nm in an ELISA plate reader.

### 2.12. Measurement of Vaccine Viral Replication by qPCR

Replication of the rPCV2-Vac virus following immunization was quantified by a TaqMan quantitative PCR (qPCR), using a SRS2 marker specific primer and probe combination and serum collected on DPV 0.14 and 28. Samples were assessed in duplicate. Viral DNA was extracted using the QiaAmp DNA mini Kit (Qiagen, Valencia, CA, USA) according to manufacturer’s instruction. Primer pairs with sequences of 5′-AAGTGGGAGGTTTGCCTTTGT-3′ and 5′-ATGGCCCAATCCTCGGAGAA-3′ and a probe with a sequence of 5′-TACCTGTTCCCCGTCGCGT-3′ were used. Briefly, 2.0 µL of extracted DNA, 0.4 μM of primers, 0.1 μM probe and a Tm of 67 °C were used in combination with the QuantiFast Probe PCR Kit (Qiagen, USA) and cycled in a qPCR thermocycler (CFX96 Touch, Bio-Rad, Hercules, CA, USA). The obtained Ct values were converted to log copy numbers using a standard curve generated with plasmid DNA encoding the SRS2 peptide marker. The specificity of the assay was evaluated using the infectious clones for the wildtype PCV2b and heterologous PCV2a and PCV2d. The lowest limit of detection of the assay was 2000 genomic copies per mL of serum.

### 2.13. Detection of Challenge Viral Replication

A qPCR assay specific to the PCV2d subtype was designed after analysis of PCV2a, PCV2b and PCV2d sequences to identify regions unique to PCV2d (Figure S1). The sequences of the primers used were 5′-GGCCTACATGGTCTACATTTCCAGT-3′ and 5′-GGTACTTTACCCCGAAACCTGTC-3′, and the probe sequence was 5′-TGGGTTGGAAGTAATCGATTGTCCTATCA-3′ (Biosearch Technologies, Novato, CA, USA). The specificity of the assay for PCV2d was evaluated by testing for the absence of detection with PCV2a and PCV2b. A standard curve was generated using cloned PCV2d genomic DNA and the lowest limit of reliable detection determined as 3000 genomic copies per mL of serum. To quantify the challenge virus loads in serum, post-challenge sera collected at DPC 9 and DPC 21 were assessed essentially as described above.

### 2.14. Assessment of Pathological Lesions

Evaluation of tissue pathology was carried out essentially as described previously [10]. Macroscopic evaluation of the major organs for gross lesions in the major organs was conducted by assessing lungs for the presence of lesions scored as the percentage of lung parenchyma affected from 1%–100%. Inguinal lymph node enlargement was scored from 0–3, where 0 was no enlargement, 1, 2 and 3 were two, three or four times the normal size. Sections of the major organs including the lung, liver, kidney, spleen ileum, tonsils, tracheobronchial and mesenteric lymph nodes were fixed in 10% buffered formalin for 48 h and then transferred to 70% ethanol for sectioning. Slides were examined by hematoxylin and eosin (H&E) staining for microscopic lesions and immunohistochemistry (IHC) to detect viral antigen, following the standard operating procedures of the Iowa State University Veterinary Diagnostic Laboratory. The slides were assigned scores ranging from 1–4 in a blinded fashion by a board-certified veterinary pathologist as follows; 1 = single follicle or focus staining, 2 = rare to scattered staining, 3 = moderate staining, 4 = strong widespread staining.

### 2.15. Statistical Analysis

A significance level of *p* <  0.05 was used for all statistical analysis. Analysis was conducted using the Minitab19 software (Minitab, State College, PA, USA) or Microsoft excel. Where data were not normally distributed, non-parametric analysis was used. Serological and qPCR data were analyzed by a Student’s *t* test. The lesion scores and body weight data were analyzed by the Mann–Whitney U test. The consolidated values, statistical significance and standard deviation are represented in the figures.

## 3. Results

### 3.1. The rPCV2-Vac Was Successfully Rescued and Expressed the Marker Peptide

**Figure 2:**
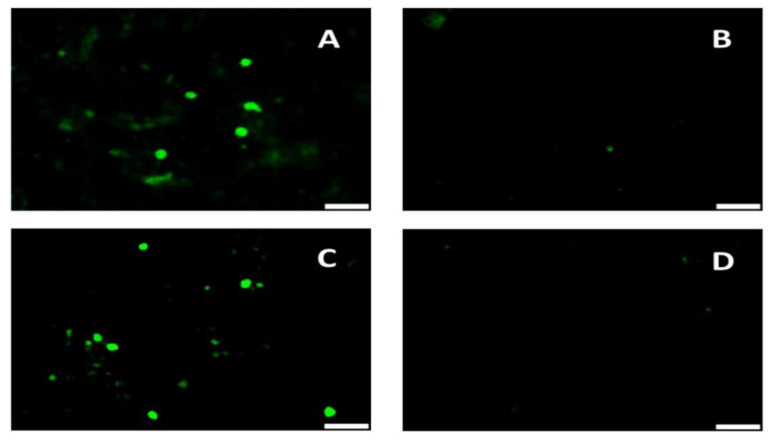
Rescue of the recombinant rPCV2-Vac. (**A**) PK-15 cells transfected with the rPCV2-Vac construct and stained with a PCV2 specific polyclonal antibody (**C**) PK-15 cells transfected with the rPCV2-Vac construct and stained with a *Neospora caninum* specific polyclonal antibody. (**B**) and (**D**) Un-transfected PK-15 cells stained with the PCV2 or *Neospora caninum* antibodies respectively. Apple green florescence is indicative of a specific signal. White bar–Scale of 50 µM. Image obtained at 20x magnification.

The reverse genetics approaches used to mutate the selected immunodominant linear B cell epitopes in the PCV2 capsid protein [7] enabled the successful rescue of the recombinant rPCV2-Vac virus (Figure 2A). Introduction of the mutations did not affect detection of the recombinant PCV2 virus by polyclonal antibodies. Expression of the marker peptide was clearly detected by a *Neospora caninum* specific antibody (Figure 2B).

### 3.2. The rPCV2-Vac Induces Binding Antibody Responses in Vaccinated Pigs

**Figure 3:**
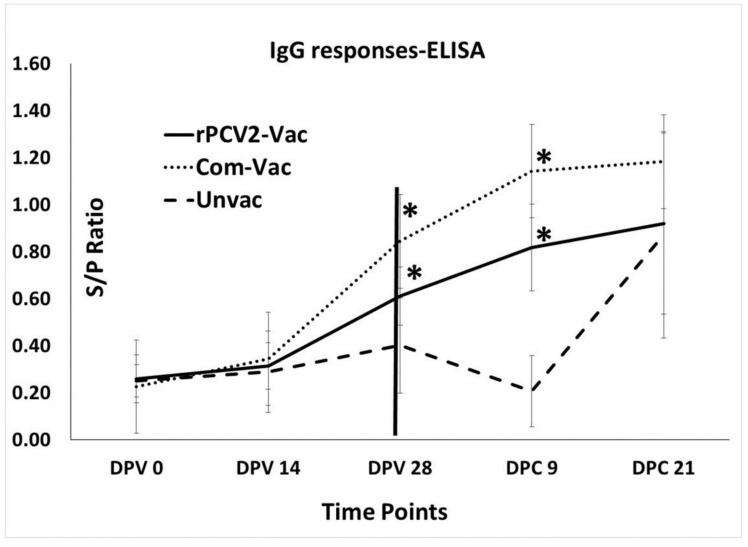
Anti-PCV2 IgG responses: Mean signal to positive (S/P) ratios of sera collected on days 0, 14 and 28 post vaccination (DPV) and on days 9- and 21-days post-challenge (DPC), as measured by a PCV2 specific commercial ELISA. X axis: time points of serum collection, Y axis: sample to positive (S/P) ratio, dotted line: commercial vaccine, solid line: rPCV2-Vac, dashed line: unvaccinated. Error bars indicate the standard deviation, * significantly different from the unvaccinated control, *p* ≤ 0.05, Students *t* test. Significant differences were not detected between the rPCV2-Vac and commercial vaccine.

Measurement of anti-PCV2 IgG responses in the study animals using a commercial PCV2 ELISA kit showed an increase in titers after 14 DPV in both the vaccine groups, with the differences between rPCV2-Vac and unvaccinated control group being significantly different at DPV 28 and DPC 09. Although a direct comparison between rPCV2-Vac and the commercial control cannot be drawn due to differences in vaccine formulation, the magnitude of the IgG response to the commercial vaccine remained consistently higher than that of the rPCV2-Vac. Antibody responses in the unvaccinated controls remained low until DPC 9, after which significant differences were not noted between the groups at DPC 21 (Figure 3).

### 3.3. The rPCV2-Vac Elicits Broad Virus Neutralization Responses

**Figure 4:**
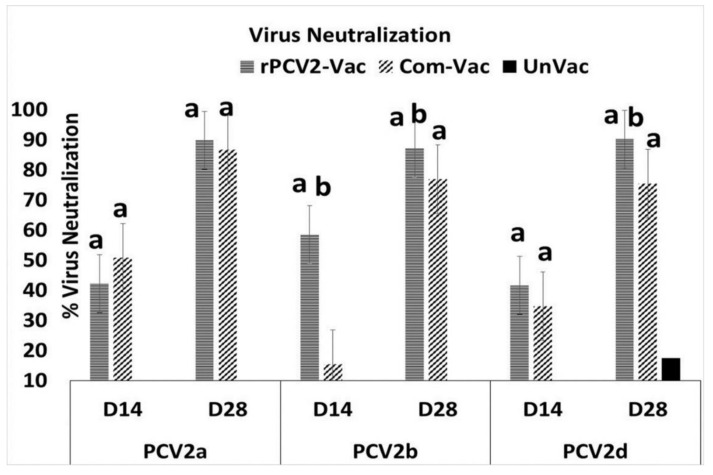
Virus neutralization responses: Mean virus neutralization titers measured by a rapid fluorescent focus reduction assay. Values for days 14 and 28 post vaccination (DPV) are depicted. No significant activity was detected in the sera of the unvaccinated control group or at day 0. X axis-virus neutralization titers against PCV2a, PCV2b or PCV2d. Y axis–% virus neutralization, horizontal lines: rPCV2-Vac, slanted lines: commercial vaccine, solid bar: unvaccinated. Error bars indicate the standard deviation, a-significantly different from the unvaccinated control, b-significantly different from the commercial vaccine group, *p* ≤ 0.05, Students *t* test.

Virus neutralizing responses were measured against the homologous PCV2b subtype, as well as heterologous PCV2a and PCV2d subtypes, using a rapid fluorescence focus reduction assay. Despite the fact that the commercial vaccine has an adjuvant and has undergone extensive dose optimization, neutralization responses elicited by the rPCV2-Vac against the PCV2a subtype were comparable in kinetics and magnitude to that of the commercial vaccine, which contains the PCV2a capsid antigen. Similarly, neutralizing responses against the currently predominant PCV2d subtype in the rPCV2-Vac group were higher than that of commercial vaccine by DPV14, with the difference becoming statistically significant at DPV28. Neutralizing responses elicited by the rPCV2-Vac against its homologous PCV2b strain were robust. However, the commercial vaccine was significantly less effective than rPCV2-Vac in neutralizing PCV2b (Figure 4).

### 3.4. Surface Plamon Resonance (SPR) Analysis of Epitope Binding

**Figure 5:**
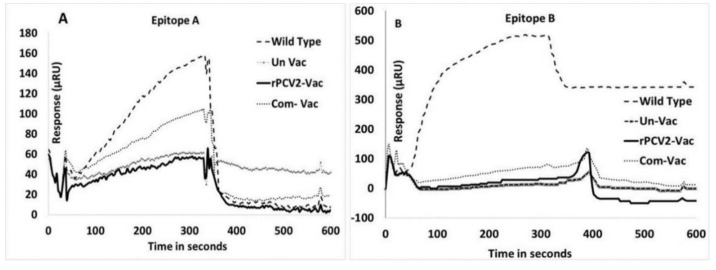
Antibody responses to the mutated epitopes: Loss of immunodominant effects due to mutation of epitopes (**A**) and (**B**) as assessed qualitatively by surface plasmon resonance. 20 µM of purified IgG was tested for all experimental antisera. X axis—Time in seconds, Y axis—Response measured in µRU (µ response units). (**A**). Responses to a peptide encoding the wildtype epitope (**A**,**B**). Responses to a peptide encoding wildtype epitope (**B**). Slashed line-anti-serum to the wildtype virus, dotted line: anti-serum to the commercial vaccine, solid line-anti-serum to the rPCV2-Vac, connected triangles: anti-serum from the unvaccinated group.

Antibody responses to epitope A and B were not detected in the serum of rPCV2-Vac immunized pigs by a qualitative SPR analysis, while the responses in pigs infected with the wildtype virus were strong. For epitope A, the response in pigs administered the rPCV2-Vac was similar to that of the unvaccinated pigs. The response in the pigs administered the commercial vaccine was of a lesser magnitude than that of the pigs infected with the wildtype virus. In the case of epitope B, strong responses were noted pigs infected with the wildtype virus, but the differences between the other three groups were not significant (Figure 5).

### 3.5. Measurement of the Marker Specific ab Responses

**Figure 6:**
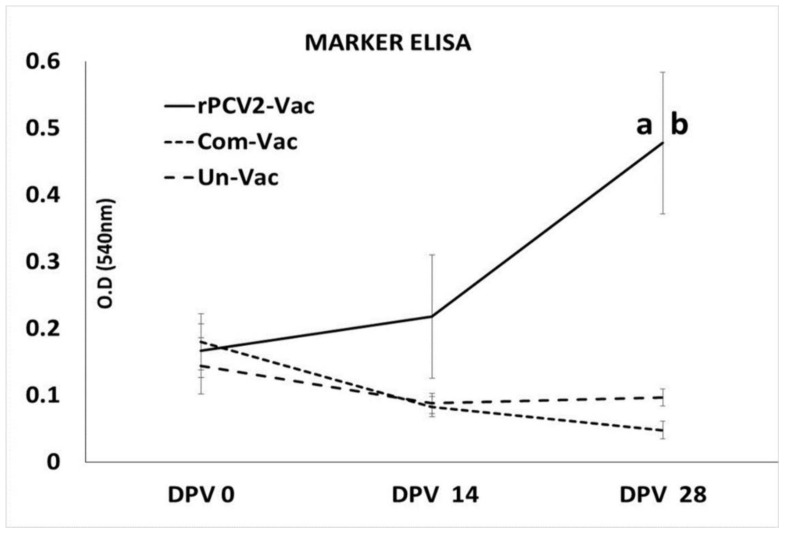
Antibody responses to the marker: Mean optical density values of sera collected on days 0, 14 and 28 post vaccination (DPV) (*N* = 9), as measured by an ELISA specific to an antigenic peptide selected from the *Neospora caninum* SRS2 protein. X axis: time points of serum collection, Y axis: mean optical density (O.D) value, solid line: rPCV2-Vac, dotted line: commercial vaccine, dashed line: unvaccinated group. Error bars indicate the standard deviation, a: significantly different from the unvaccinated control, b: significantly different from the commercial vaccine group; *p* ≤ 0.05; Students *t* test.

Assessment of the antibody responses to the marker by an ELISA specific to the peptide selected from the *N. caninum* SRS2 protein showed that pigs vaccinated with the rPCV2-Vac mounted detectable Abs responses to the marker by DPV14, with the responses becoming significantly different from not only the unvaccinated control group but also the commercial vaccine by DPV 28. The unvaccinated pigs and pigs administered the commercial vaccine did not mount significant antibody responses to the marker (Figure 6).

### 3.6. Vaccination Protects Against Challenge Viral Replication

**Figure 7:**
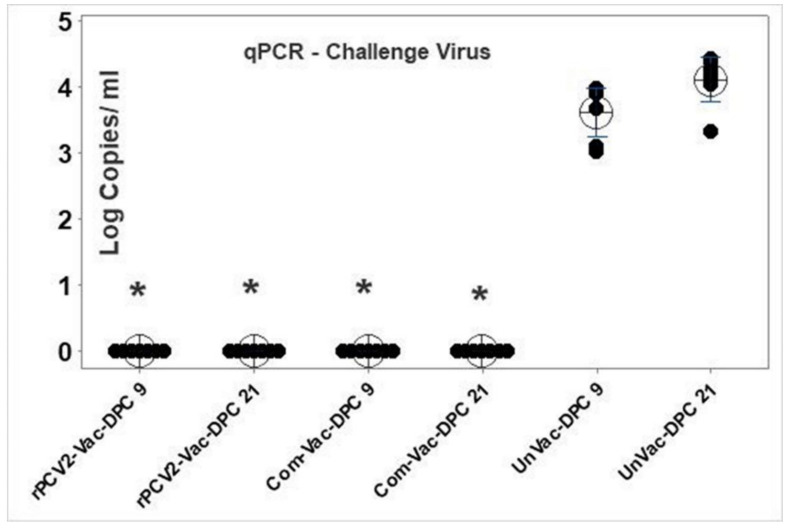
Replication of the challenge virus. Quantification of the heterologous PCV2d challenge virus at 9 and 21 days post challenge by a PCV2d subtype specific qPCR. X axis: experimental groups and time of serum collection, Y axis: log_10_ viral copy numbers per mL of serum. Interval bars: 95% confidence interval of the means. * Significantly different from the unvaccinated control group; *p* ≤ 0.05; Students *t* test.

Replication of the heterologous PCV2d challenge virus was not detected in the sera either of the vaccine groups at DPC 9 or DPC 21. Robust challenge viral replication was detected in the unvaccinated pigs, with the viral titers increasing by about 1 log between day 9 and day 21 post-challenge. The values for both vaccine groups were significantly different from the unvaccinated control group at both the time points tested (Figure 7).

In contrast to wildtype PCV2 viruses, which can be easily detected by qPCR by DPC 9 (Figure 7), viremia due to the rPCV2-Vac virus was not detected by the SRS2 tag-specific qPCR assay in the sera of any of the vaccinated pigs at DPV14. The rPCV2-Vac virus was detected at low levels in the serum of only one out of nine pigs at DPV 28, indicating that the rPCV2-Vac was attenuated in vivo. Sequencing of the rPCV2-Vac genome from the viremic pig confirmed the presence of the mutations in the two epitopes and the presence of the SRS2 marker, indicating the vaccine remained stable in the host. Similarly, sequencing of the rPCV2-Vac genome after three passages in cell culture showed that the mutated and inserted sequences were intact, suggesting that the vaccine was genetically stable in vitro.

### 3.7. Protection against Gross and Histological Lesions

**Figure 8:**
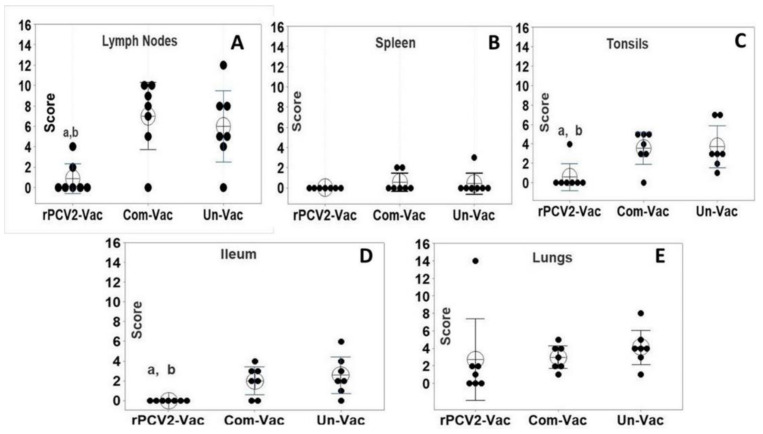
Lesion scores in tissues. Assessment of lesion scores resulting from viral challenge is represented as the sum of the scores for each tissue (**A**–**E**). Gross lung lesions were scored from 0%–100% to represent the % area of affected lung. Microscopic lesions were scored with a scale of 1–4; where 1 = single follicle or focus staining 2 = rare to scattered staining, 3 = moderate staining 4 = strong widespread staining. X axis: groups, Y axis: scores, dots: values for the individual pigs, horizontal bar with the large circle: group mean, bars: 95% confidence interval of the means, a: Significantly different from the unvaccinated control, b: Significantly different from the commercial vaccine group, (*p* < 0.05) by a Mann–Whitney U test.

Except for the lungs, gross lesions were not observed in any of the other major organs for all experimentally challenged pigs. For the lymph nodes, the microscopic lesion scores (consisting of the sum of the H&E and IHC scores), were significantly lower for the rPCV2-Vac group than those of the commercial vaccine group and the unvaccinated group (Figure 8A) with only two out of seven pigs showed mild changes, while six of seven the pigs in the control groups showed histiocytic infiltration and lymphoid depletion. Microscopic lesions were not detected in the spleen (Figure 8B) liver and heart. The microscopic lesion scores of the ileum and tonsils (Figure 8C,D) of the rPCV2-Vac group were also significantly lower than that of the control groups. The pulmonary lesion scores in the rPCV2-Vac group were lower than that of the controls but the difference was not statistically significant (Figure 8E). The overall lesion scores for the rPCV2-Vac was highly significantly different from the control groups (Figure 8F), while the scores of the commercial vaccine group was similar to that of the unvaccinated group. Significant gross or microscopic lesions were not observed in the pigs sacrificed prior to challenge (two pigs per group) to assess vaccine safety. There were no significant differences in the lesion scores between the experimental groups, indicating that the rPCV2-Vac was both attenuated and safe.

### 3.8. Vaccination Protects against Weight Loss Due to Challenge

As is commonly encountered in experimental models, severe clinical signs of PCVAD were not observed in any of the experimental groups during the 21 days post-challenge observation period. However, the post-challenge weight gain in both vaccination groups were significantly higher than the unvaccinated control group at DPC 21 (Figure S3B), but not at DPC 14 (Figure S3A). There were no significant differences between the two vaccine groups during the post-challenge observation period (Figure S3).

## 4. Discussion

The phenomenon of “original antigenic sin” or ability to elicit memory responses to antigens and specific epitopes is critical to the success of vaccination. On the other hand, the preferential clonal expansion to immuno-dominant but non-protective epitopes encountered by the host on challenge, coupled with minor sequence variation leading to escape variants, is an elegant immuno-subversion strategy we term “deceptive imprinting”. Strategies to counter deceptive imprinting in vaccine design include “dampening” the response to the immuno-dominant non-protective epitopes [5]. The immune refocusing strategy has been successfully applied to several viruses, such as human immunodeficiency virus (HIV) [18,19], influenza [20,21] and dengue virus [22], among others. Unlike structurally complex pathogens, where protection is mediated by multiple antigens, the requirement for a single protective antigen makes PCV2 both a simple and elegant model for studying the effects of immunodominance on vaccine design. In this study we explored the hypothesis that alteration of the immunodominance properties of the PCV2 capsid protein will enhance rational vaccine design and result in significant protection against challenge.

The PCV2 capsid protein contains four major immunodominant regions [23]. Within these regions, four putative immunodominant non-protective linear B cell epitopes were identified [7,8]. As the PCV2 capsid protein is relatively small (233 amino acids), and incapable of tolerating large sequence changes, only two of the identified decoy epitopes were selected for mutation in this study. It was previously demonstrated that mutation of an immunodominant HIV-1 epitope located in proximity to a neutralizing epitope can direct the response towards the neutralizing epitopes, possibly due to alteration of steric constraints [24]. As both epitope A and B were flanked by putative neutralizing epitopes [7] they were selected for analysis. To minimize the risk of introducing lethal mutations, we elected not to delete residues, but rather replace them with other residues with a low penalty score on a point accepted mutation (PAM) matrix [11,25], and were able to successfully rescue the recombinant virus harboring mutations in the selected epitopes (Figure 2).

As anticipated, the introduced changes to the amino acid sequences of the PCV2 capsid protein resulted in the loss of immunodominance of epitope A and B as assessed by SPR (Figure 5). As paratopes which bind rapidly to their epitopes receive stronger stimulatory signals and can influence the magnitude of clonal expansion during the affinity maturation stage [26,27], an assessment of the affinity kinetics of the Abs generated in this study to their cognate peptides or to peptides encoding the mutations could not be carried out due to a shortage of samples, and only a qualitative measurement was obtained by SPR (Figure 5). Interestingly, antibody responses to epitope B were not detected in pigs administered the commercial PCV2 vaccine. It has been previously suggested that vaccination with fully assembled viral particles does not induce strong Ab responses to epitope B while vaccination with monomers of the subunit does [8]. Further, MHC-II processing for the same antigen is known to differ between endogenous and exogenous antigens which may be introduced by infection or vaccination respectively [15,28]. A limitation of this study is that only linear epitopes were targeted.

Several other factors, such as glycosylation, hypervariability, proximity to MHC-II epitopes or other neutralizing epitopes, could also potentially influence the outcomes of this study. While a detailed experimental characterization of the above listed parameters is not within the scope of the study, they are discussed below. Hyper-glycosylation is a strategy which has been previously used to dampen the Ab response to immunodominant epitopes [29]. While not the primary strategy targeted in this study, the alteration in glycosylation patterns as described in the method section could have influenced the outcomes of this study. As immunodominance is influenced by the successful competition for the recruitment of antigen specific T cells in early infection, the presence of a helper T cell epitopes overlapping or adjacent to a B cell epitope can influence the strength of the Ab response elicited, [30]. Epitope A contained a predicted (Propred MHC-II server) [31], but non-conserved, MHC-II epitope 124 ILDDNFVT31 [32], which was altered by the mutation of the residue T to an N. Two conserved predicted MHC-II epitopes, 161 FTPKPVL167 and 174 FQPNNKRNQL184 overlapped with epitope B [32]. The second predicted MHC-II epitope within epitope B was also altered by the mutations introduced. It is possible that mutation of these T helper epitopes could have enhanced the loss of immunodominance of Epitopes A and B.

Hypervariability is a common property of decoy epitopes [5], and is an effective immuno-subversion mechanism. However, Epitope A and B were conserved between the first discovered PCV2a and PCV2b subtypes (Table S1, Figure S1). Only residue 131 in epitope A and residue 169 in epitope B varied between the newly evolved PCV2d challenge strain and the previously existing PCV2a and 2b subtypes (Table S1, Figure S1). For influenza, it has been suggested that the reduced vaccine efficacy observed for the H3N2 component of the polyvalent vaccine could result from the reinforcement of persistent and preferential strain specific memory (deceptive imprinting) to the H1 subtype and B type by annual vaccination, leading to competition between the polyvalent antigens [33]. Therefore, prior exposure to the unmodified epitopes A and B by infection with PCV2a or 2b, or by vaccination, could diminish protection against the newly evolved PCV2d subtype in the field [34,35]. While direct comparisons of the rPCV2-Vac to the commercial control vaccine are avoided as the commercial vaccine is extensively standardized for optimal dosage which can differ from the experimental vaccine, is inactivated and contains an adjuvant, in this study, the rPCV2-Vac was significantly more effective at inducing neutralizing Ab responses against the heterologous PCV2d subtype (Figure 4).

While vaccine viral replication was not detected at 14 days post-vaccination, it is possible that replication of the experimental vaccine virus could have been detected if sampling was done at time points prior to day 14. However, the fact that vaccine virus was not detected at day 14 while wildtype viruses increase in titers at 14 days post-infection supports the conclusion that rPCV2-Vac was attenuated. The broadened virus neutralization responses elicited by vaccination with rPCV2-Vac (Figure 4) correlated with the significant reduction in tissue pathology caused by early challenge viral replication and localization to the sites of predilection (Figure 8). The reduced lesion scores in lymphoid organs, which are the primary sites of predilection for PCV2, indicate the rPCV2-Vac was highly effective in curtailing local infection as well as systemic dissemination. Overall, the data supports the conclusion that rPCV2-Vac was more effective in neutralizing heterologous subtypes than the PCV2a based commercial vaccine, while acknowledging that the observed effects could be due to differences in the nature of the treatments, as the commercial vaccine is an inactivated preparation and the rPCV2-Vac is a live virus (Figure 4).

The exact mechanisms by which a low-level of exposure to protective antigens is successful in eliciting good vaccine efficacy is not fully understood. However, initial priming of the immune response is known to be critical in influencing the quality of the response. Recent studies in cancer immunotherapy have shown that low doses of antigen, rather than high doses, preferentially primed high avidity CD4^+^T cells, which in turn stimulated both antibody [8] responses and cytotoxic T cell responses effectively, instead of skewing the response towards one arm of the immune system [36]. While it is possible that the insertion of the SRS peptide marker at the 5′end of the capsid gene could have influenced outcomes, the presence of the tag itself if unlikely to provide PCV2-specifc immunity or enhance protection. It has been reported that PCV2 can be detected in the nasal secretions in the absence of viremia, post-challenge [37]. However, a limitation of this study is that shedding of the challenge virus in nasal secretions or fecal matter was not measured. With the reasonably strong performance of current PCV2 vaccines in the field, the availability of an enhanced vaccine could pave the way for the eventual eradication of the virus [1]. Successful disease eradication efforts in veterinary medicine typically employ a stamping out strategy, wherein infected animals can be differentiated from vaccinated animals using serological assays and then removed from the herd in a systematic manner [38]. Detection of antibody responses to the SRS2 peptide will only provide information regarding whether an animal is vaccinated and will not enable differentiation of animals which can get infected after they receive the vaccine. However, availability of the SRS2 tag enables the monitoring of vaccine compliance in the field (Figure 2 and Figure 6). With additional dose optimization and possible commercialization, the improved efficacy parameters of the rPCV2-Vac could reduce or eliminate the emergence of new PCV2 subtypes, and significantly advance current control measures for PCV2.

## 5. Conclusions

Thus, targeted modification of the selected non-protective immunodominant epitopes in the PCV2 capsid protein resulted in broadened virus neutralization responses against newly evolved heterologous PCV2 strains, prevented replication of the challenge virus and development of tissue pathology in vaccinated and challenged pigs. The described approach can potentially have a broad application in rationalizing vaccine design for agents with delayed virus neutralizing antibody responses.

## 6. Patents

The submitted work is protected by a provisional patent application, in compliance with the regulations of North Dakota State University.

## Supplementary Materials

The following are available online at https://www.mdpi.com/2076-393X/8/3/506/s1. Figure S1: Multiple sequence alignment of the PCV2 capsid protein, Figure S2: Map of the rPCV2-Vac construct, Figure S3: Post-challenge weight gain, Table S1: Amino acid sequences of Epitope A and B.
