# Supplementary material for: Targeted Alteration of Antibody-Based Immunodominance Enhances the Heterosubtypic Immunity of an Experimental PCV2 Vaccine
Source: Vaccines (Basel). 2020 Sep 4;8(3):506. doi: 10.3390/vaccines8030506 (PMC7563983; doi:10.3390/vaccines8030506)
Supplement: Supplementary file 1 [file vaccines-08-00506-s001.zip › vaccines-883395-supplementary.pdf]

Table S1. Amino acid sequences of Epitope A and B.

Subtype	Epitope A	Epitope B
PCV2a (AF264042.1)	124 ILDDNFVTKATALTYDPY 141	166 VLDSTIDYFQPNNKR 180
PCV2b (KR816332)	124 ILDDNFVTKATALTYDPY 141	166 VLDSTIDYFQPNNKR 180
rPCV2-Vac	124 ILDDNFVNKSTALTYDPY 141	166 VLDSTIDYFNPNNSR 180
PCV2d (JX535296.1)	124ILDDNFVTKANALTYDPY141	166 VLDRTIDYFQPNNKR 180

Shadowed residues-mismatches from the PCV2b vaccine (KR816332) backbone, Residues in a larger font size–residues mutated in the rPCV2-Vac, Underlined residues–putative glycosylation sites (NetNGlyc 1.0 Server, DTU Bioinformatics, Department of Bio and Health Informatics, Copenhagen, Denmark).

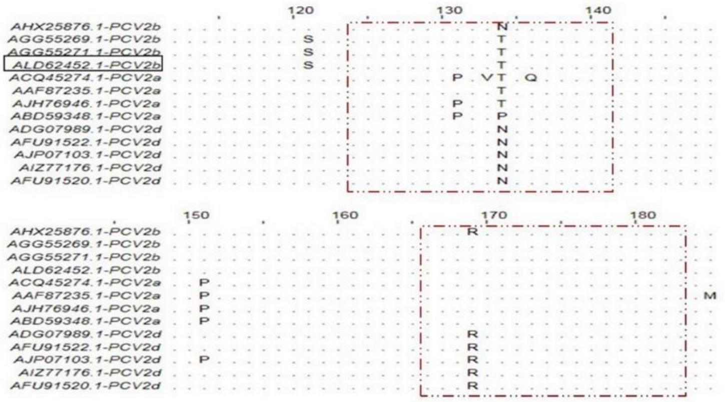

Figure S1. Multiple sequence alignment of the PCV2 capsid protein: Selected amino acid sequences of the PCV2 capsid protein representing the major circulating subtypes PCV2a, b and d, generated using the Jal View 2.4 software (The Barton Group, Dundee, Scotland, UK). Conserved residues are indicated by dots. Boxes represent epitope A and B. The boxed accession number pertains to the rPCV2-Vac backbone.

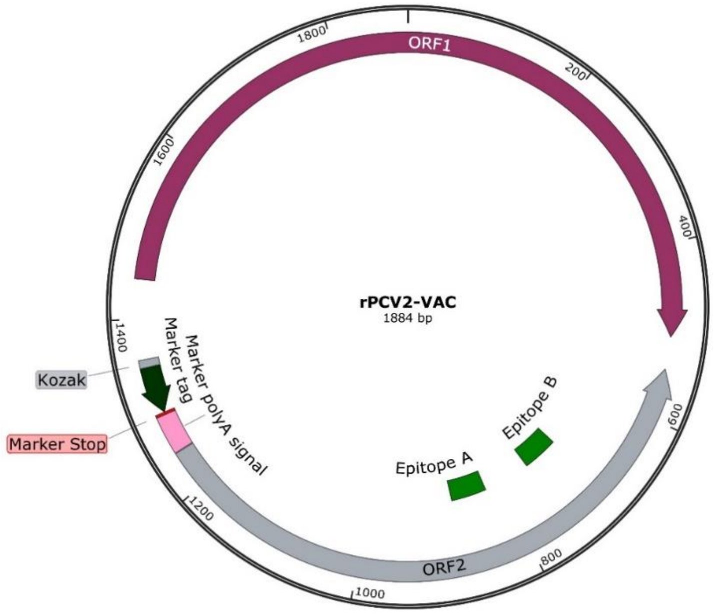

Figure S2. Map of the rPCV2-Vac construct: Diagrammatic representation of the PCV2b infectious clone showing the PCV2b genome, major open reading frames, location of Epitope A and B and the insertion site of the marker tag as an independent transcriptional unit in the 5′ end of the capsid gene (ORF2).

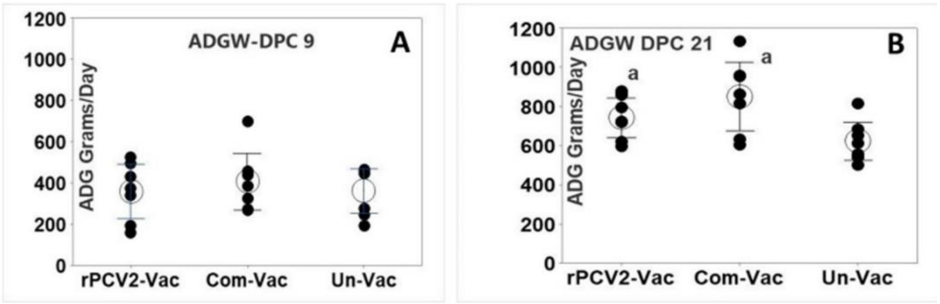

Figure S3. Post-challenge weight gain: The effects of heterologous viral challenge on the weight gain of vaccinated pigs is depicted as the average daily gain in weight (ADGW) in grams/day. (**A**). Weight gain of the experimental pigs on DPC 9, (**B**)—Weight gain of the experimental pigs on DPC 21. X axis: Groups, Y axis: ADG grams/day, horizontal bar with the large circle: group mean, bars: 95% confidence interval of the means, a: significantly different from the unvaccinated control, ($p < 0.05$) by the Mann–Whitney U test.
